# Nitrogen dynamics along a climate gradient on geologically old substrate, Kaua’i, Hawai’i

**DOI:** 10.1007/s00442-018-4285-1

**Published:** 2018-10-30

**Authors:** Peter M. Vitousek, Elizabeth L. Paulus, Oliver A. Chadwick

**Affiliations:** 1 Department of Biology, Stanford University, Stanford, CA 94305 USA; 2 Department of Geography, University of California, Santa Barbara, Santa Barbara, CA 93106 USA

**Keywords:** Soil nutrients, Pedogenic thresholds, δ^15^N, Mineralization, Nitrification

## Abstract

We evaluated N dynamics on a climate gradient on old (> 4 million year) basaltic substrate on the Island of Kaua’i, Hawai’i, to evaluate the utility of pedogenic thresholds and soil process domains for understanding N cycling in terrestrial ecosystems. Studies of nitrogen dynamics on the climate gradient on a younger basaltic substrate (~ 150,000 year) had found a good match between soil process domains and N cycling processes. Here we measured net N mineralization and nitrification by incubation, and δ^15^N of total soil N, to determine whether the soil process domains on the older gradient were equally useful for interpreting N cycling and thereby to explore the general utility of the approach. Net N mineralization varied from 0 to 1.7 mg kg^−1^ d^−1^ across the old Kaua’i gradient, and δ^15^N varied from + 3 to + 11 ^ο^/_οο_, both ranges similar to those on the younger substrate. However, while the pattern of variation with climate was similar for δ^15^N, the highest rates of mineralization on the old gradient occurred where forests were dominated by the native N fixer *Acacia koa*. This occurred in sites wetter than the process domain associated with high net N mineralization on the gradient on younger substrate. We conclude that soil process domains based on rock-derived nutrients are not always useful for evaluating N dynamics, especially where the distribution of biological N fixers is controlled by factors other than rock-derived nutrients.

## Introduction

Nitrogen (N) is the element that most often limits primary production and other ecosystem processes in little-managed ecosystems, either alone or in combination with other elements (frequently phosphorus) (Elser et al. 2007; Hogberg et al. 2017). Despite its importance, N is rarely an independent control over ecosystems, in the sense that (absent fertilization, anthropogenic atmospheric deposition, or other interventions) the pools and availability of N reflect the influence of other controlling processes such as biological N fixation and its controls (often including the availability of phosphorus), and the losses of N by pathways that are dependent upon versus independent of the demand for N within ecosystems (von Sperber et al. 2017).

Recent research in the Hawaiian archipelago and elsewhere has identified pedogenic thresholds and soil process domains that emerge along continuous gradients of environmental forcing. Pedogenic thresholds occur where soil properties or processes change abruptly and/or non-linearly along a continuous gradient, while soil process domains are the regions between thresholds where soil properties and processes change relatively little despite a large range of environmental forcing (Muhs 1984; Chadwick and Chorover 2001; Vitousek and Chadwick 2013). Despite the importance of N to biological systems, analyses of thresholds and domains have not generally included N—at least in part because the approach derives from pedology, and pedologists generally consider N to reflect other, independent controlling processes rather than itself being an independent control of soil and ecosystem dynamics.

Recently, von Sperber et al. (2017) evaluated the connections between thresholds and domains and the dynamics of N along a climate gradient on Kohala Volcano on the Island of Hawai’i, exploring whether thresholds and domains could provide insight into the controls of N pools and processes. They found that N availability was low (though soil organic N pools were large) in a domain of high-rainfall, acid, infertile soils in which the potential for inputs of nutrients via mineral weathering was exhausted, that N availability was high in an intermediate-rainfall domain dominated by ongoing weathering and biological uplift of non-N nutrients, and that N availability was low (as were N pools) in a dry domain in which weathering (as well as N mineralization) was constrained by low water availability. They speculated that biological N fixation was enhanced by high levels of P and Ca availability in the middle domain, increasing N availability and transformations there, but that N availability was suppressed by constraints to N fixation associated with low availability of rock-derived nutrients (and by losses of N via dissolved organic N) in the wetter domain and by unavoidable gaseous losses of N, some occurring when dry soils are re-wetted, in the drier domain. Consistent with this speculation, the natural abundance of ^15^N became strongly enriched (to > + 14 ^ο^/_οο_) in the dry domain, likely as a consequence of fractionating gaseous losses in the drier sites.

In this paper, we evaluate aspects of N cycling and availability along a climate gradient on much older substrate on the Island of Kaua’i (> 4 million years, vs. ~ 150,000 year for the climate gradient on Hawai’i Island) to evaluate the generality of the results in von Sperber et al. (2017). Earlier work on non-N soil properties on this older gradient demonstrated that pedogenic thresholds (and consequently the domains between them) were shifted to lower rainfall compared to their position on the younger gradient, a consequence of cumulative weathering, a predominance of crystalline secondary minerals (versus highly reactive and retentive poorly crystalline minerals), and millions of years of leaching on the older substrate (Chadwick et al. 2003; Vitousek and Chadwick 2013; Vitousek et al. 2016). This shift in soil process domains allows us to disentangle any direct effects of climate from consequences of soil process domains in determining patterns and putative controls of N dynamics.

## Methods

### Sites

The Hawaiian Islands are useful for understanding controls of N cycling because many of the ultimate factors that influence soils can be held constant there to a much greater extent than is possible in most continental settings (Chadwick et al. 2003; Vitousek 2004). The parent material in which soils form is basalt, varying from tholeiitic basalt erupted during the shield-building stage of a volcano’s evolution to alkalic basalt produced later in its life (MacDonald et al. 1983). The constructional surface of shield volcanoes supports little topographic variation, and remnants of those constructional surfaces can be identified on the oldest high islands. Even the dominant organisms are (relatively) consistent; the few plant species that dispersed naturally to Hawai’i have radiated to occupy a much wider range of environments than is typical in continental systems. In contrast, substrate age and climate vary widely, but generally in well-defined and continuous ways. Lava flow and other surface ages are well constrained and vary nearly continuously from recent deposits to almost 5 million years old from southeast to northwest across the archipelago. Temperature varies with elevation, and current precipitation varies predictably with aspect and elevation from < 250 to > 10,000 mm year^−1^.

While climate has varied through the history of the archipelago (Hotchkiss et al. 2000) and Hawaiian soils carry the imprint of past as well as present climate (they are polygenetic, in the sense of Richter and Yaalon 2012), the enduring characteristics determining rainfall patterns in Hawai’i are interactions of the Northeast trade winds with island topography. The direction of the trade winds has been consistent for several million years (Porter 1979). Consequently, dry sites on modern rainfall gradients have long received less rainfall than wet sites, and older sites have accumulated more rainfall (and leaching) than younger sites (Hotchkiss et al. 2000).

The climate gradient that we sampled on Kaua’i extended from the Alaka’i Swamp on the wet extreme (the wettest site received > 3800 mm year^−1^ of precipitation) to sites in western Kaua’i receiving ~ 600 mm year^−1^ of precipitation (Giambelluca et al. 2013). While temperature varied from ~ 19 °C in the driest sites to 16 °C in the wettest (Giambelluca et al. 2014), and was closely (and inversely) correlated to annual precipitation, we believe variation in precipitation to be the dominant control of ecosystem properties on this gradient and the younger gradient to which we compare the Kaua’i gradient (von Sperber et al. 2017). Rainfall varies much more substantially than does temperature on these gradients, and (more broadly across Hawai’i) ecosystems with similar average temperatures but different precipitation have structures and compositions that differ more than those of ecosystems with similar precipitation but different average temperatures (Vitousek et al. 1992; Aplet et al. 1998).

All of the sites we used were located on the constructional surface of the Kaua’i volcano. Erosion associated with Waimea Canyon made it difficult to find sites on constructional surfaces receiving from 1000 to 1500 mm year^−1^ of precipitation. Land use ranged from grazed sites dominated by introduced grasses and woody vegetation (some of it native) on the dry end of the gradient to mesic and wet native-dominated forest on the wetter end. Climatic parameters were obtained from Giambelluca et al. (2013, 2014). Soils on the gradient ranged from Haplotorrox, Typic Eutrotorrox, and Tropeptic Haplustox in the driest sites through Haplustox and Plinthic Acrorthox to Typic Gibbsihumox and ultimately a Humic Epiaquept in the highest-rainfall site (Foote et al. 1972). These soil classes illustrate a key difference between the Kaua’i soil gradient and the one on Kohala. On Kaua’i, the Oxisols are dominated by crystalline clays that have relatively low nutrient holding capacity, whereas on Kohala soils are Andisols dominated by metastable and highly reactive short-range-order minerals that can retain considerable quantities of nutrients and carbon (Torn et al. 1997).

### Soil sampling

Soils were collected as a continuous sample from the surface to 30-cm depth on two occasions, with different (but overlapping) measurements each time. In the first sampling in 2011, we collected 33 30-cm samples in upland slope positions across the climate gradient. Five of the samples in the center of this gradient were near a canyon rim and had evidence of erosion; these were discarded, and we based our analyses on the other 28. In the second sampling (2017), which was focused on N, we collected 18 30-cm samples across the gradient, avoiding areas where erosion had influenced soil properties. Finally, we compared results from Kaua’i with those from Kohala Volcano on the Island of Hawai’i, where we had collected and analyzed 30-cm samples from 160 sites arrayed across a climate gradient for non-N soil properties and from 46 sites on the same gradient for N transformations and processes. During soil sampling, we noted the occurrence of potential N fixers near the sampling site on both gradients; on Kaua’i, two sites in each sampling campaign were strongly dominated by the native N-fixing tree *Acacia koa*.

The 30-cm samples allow extensive spatial analyses, but exclude deeper soil properties and feedbacks between surface and subsurface processes. Accordingly, we drew upon information from complete soil profiles that had been sampled by horizon. This sampling included 6 sites arrayed along the Kaua’i climate gradient; soils were sampled from hand-excavated pits, supplemented by deeper sampling (to 4-m depth) with an auger, and in one case sampling to 17-m depth from a cliff face exposed by recent erosion (Vitousek and Chadwick 2013). These deeper soils are more important for rock-derived nutrients than for N, since organic N and organic N turnover are heavily concentrated in surface soils.

### Soil analyses

Soils from the first sampling in 2011 were air-dried, passed through a 2-mm sieve, divided into three homogenous subsamples, and analyzed as described in the supplemental material to Vitousek et al. (2004); results are reported in Vitousek and Chadwick (2013). Briefly, one subsample was analyzed for resin-extractable phosphorus (P) and for total carbon (C) and N at Stanford University; P was determined using the anion-exchange resin method of Kuo (1996) and analyzed on an Alpkem RFA/2 AutoAnalyzer, while total C and N were analyzed using a Carlo Erba NA 1500 elemental analyzer. A second subsample was analyzed for pH and cation exchange capacity (CEC) and exchangeable Ca, Mg, Na, and K at the University of California, Santa Barbara, using the NH_4_OAc method at pH 7.0 (Lavkulich 1981). We measured CEC buffered at pH 7 and used it in the calculation of base saturation as a way to standardize our measurements among mineralogically diverse soils. The third subsample was shipped to ALS Chemex (Sparks, Nevada, USA) and analyzed for total concentrations of 11 elements (Si, Al, Fe, Ca, Mg, Na, K, Ti, P, Nb, Zr) using lithium borate fusion followed by X-ray fluorescence spectrometry. Finally, soil δ^15^N (defined as the parts per thousand difference between the sample and a consistent standard) was analyzed in the Stable Isotope Biogeochemistry Laboratory at Stanford University (https://earthsci.stanford.edu/research/sibl/), using a Carlo Erba CN Analyzer coupled to a Thermo Finnigan Delta Plus mass spectrometer.

The second set of soils (collected in 2017) was analyzed for potential net nitrogen mineralization and nitrification and natural abundance δ^15^N, largely following the procedures in von Sperber et al. (2017). For potential net N mineralization, we weighed 10 g (wet weight) of each soil sample into each of three plastic cups. One cup was extracted immediately by shaking with 100 ml of 2 N KCl and collecting (pipetting or when necessary filtering with 0.2 μM syringe filters) a 3-ml sample for analysis of initial ammonium- and nitrate-N concentrations. Another cup of each soil was covered with a plastic cap and incubated for 3 weeks at 22 °C, adjusted back to its initial (field) water content every 3 days. These samples were then extracted with 2 N KCl as described above. For the third subsample, we adjusted water content to a constant 45% (water content over soil dry mass) for all of the soils that were drier than this value in the field, and we allowed the wetter soils to dry towards 45% through the 3-week incubation. This 45% value was selected as being close to field capacity of these soils and close to the field moisture content in moderately high-rainfall sites on the gradient. These soils were adjusted back to 45% water content every 3 days and extracted in 2 N KCl after 3 weeks. We calculated potential net N mineralization as $[({\text{final ammonium}} + {\text{nitrate}} - {\text{N}}){-}({\text{initial ammonium}} + {\text{nitrate}} - {\text{N}})]/{\text{days of incubation}}$; net nitrification was calculated as $[({\text{final nitrate}} - {\text{N}}){-}({\text{initial nitrate}} - {\text{N}})]/{\text{days of incubation}}$; our several-day refrigerated storage of field-collected samples may have caused “initial” values to differ from “field” values (Turner and Romero 2009) but would not have affected the calculation of net mineralization. Potential net N mineralization of samples incubated at field moisture content are described as ambient; those at 45% water content are described as adjusted. Ammonium- and nitrate-N were analyzed using a WestCo Smartchem Discrete Analyzer 200 in the Environmental Measurements 1 (EM-1) Laboratory at Stanford University (http://em1.stanford.edu/); soil δ^15^N was analyzed as described above.

### Calculations

**Fig. 1:**
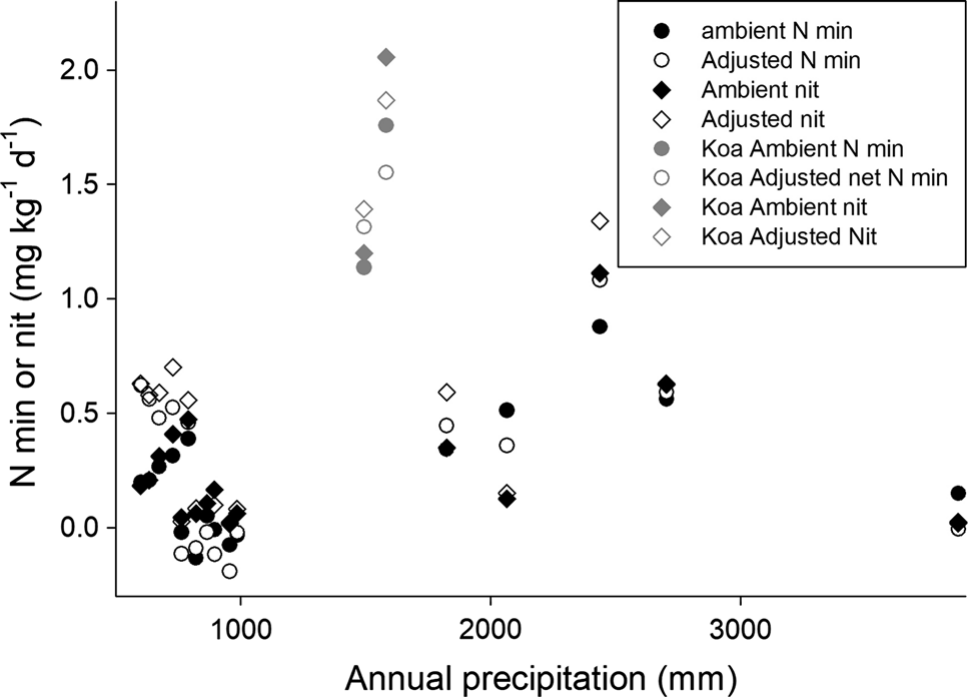
Net nitrogen mineralization and nitrification at ambient (field) soil moisture content and with soil moisture content adjusted to 45%, as a function of precipitation, for sites across the Kaua’i climate gradient. Sites dominated by the native N-fixer *Acacia koa* are shown in gray

We summarize patterns of variation along the gradient by plotting measured or calculated values versus rainfall (from Giambelluca et al. 2013). The influence of water on biogeochemical processes could be presented in terms of water deficit or excess [precipitation minus potential evapotranspiration (PET)], but variation among sites and between gradients in PET was small (Fig. 1), non-linear, and variations were not readily interpretable (Giambelluca et al. 2014), so we simply used rainfall to characterize both of the gradients. Cloudwater inputs are not included in Giambelluca et al. (2013); including them would have little effect in the dry sites but would increase inputs in the wet sites substantially.

## Results

Characteristics of soils on the Kaua’i gradient other than those related to N were reported previously (Vitousek and Chadwick 2013). Briefly, pedogenic thresholds that bounded soil process domains were shifted to drier positions on the much older Kaua’i substrate than on the Kohala substrate. That pattern applied to both highly mobile and less mobile elements and to the top 30 cm of soil and to deep soils (Vitousek and Chadwick 2013). We determined the weathering and loss of the relatively immobile element P from the ratio of concentrations of total P to those of the immobile index element Nb—concentrations of both P and Nb differ in the eruptive phases of Hawaiian volcanoes (tholeiitic versus alkali) (Garcia et al. 2010), but the ratio of P to Nb in those parent materials varies much less than do concentrations (ratios of 72 and 92 for tholeiitic basalt and alkalic basalt, respectively, versus 3.5- to 4.5-fold for concentrations). We found that wetter sites had lost much more P than had drier sites on the gradients (Vitousek and Chadwick 2013), and the transition from high to low P in dry versus wet sites occurred in wetter portions of the Kohala than the Kaua’i gradient. Weathering and loss of P was nearly complete by an average annual rainfall of 1500 mm year^−1^ (average water balance of 0) for the Kaua’i gradient, while some parent material P was retained in Kohala sites to rainfall of > 2000 mm year^−1^ (Vitousek and Chadwick 2013). Forest fertilization experiments in one site receiving ~ 2500 mm year^−1^ of precipitation on the Kaua’i gradient demonstrated that P supply limits primary production there (Herbert and Fownes 1995; Harrington et al. 2001). Exchangeable Ca increased near 2000 mm year^−1^ annual precipitation and peaked just below 1500 mm year^−1^ (a water balance of 0) on the Kohala gradient, but did not begin to increase until below 1000 mm year^−1^ on the Kaua’i gradient and was at its highest level in the driest site (Vitousek and Chadwick 2013). Soil pH increased from ~ 4 to ~ 6 from 1500 to 600 mm year^−1^ annual precipitation on the Kaua’i gradient and from ~ 4 to ~ 6 from 2200 to 1000 mm year^−1^ annual precipitation on the Kohala gradient (Vitousek and Chadwick 2013).

### N transformations and N pools

Net N mineralization and nitrification at ambient and adjusted water content did not differ systematically except in the driest sites, where soil water content ranged between 21% and 29% at the time soils were collected and mineralization and nitrification were water-limited at field moisture (Fig. 1). The highest rates of mineralization and nitrification occurred in two sites with precipitation ~ 1500 mm year^−1^ (water balances close to 0); these were the only two sites sampled that were dominated by the native N-fixing tree *Acacia koa*. Low rates of net N mineralization and nitrification occurred in the wettest site on the gradient and in many of the dry sites.

**Fig. 2:**
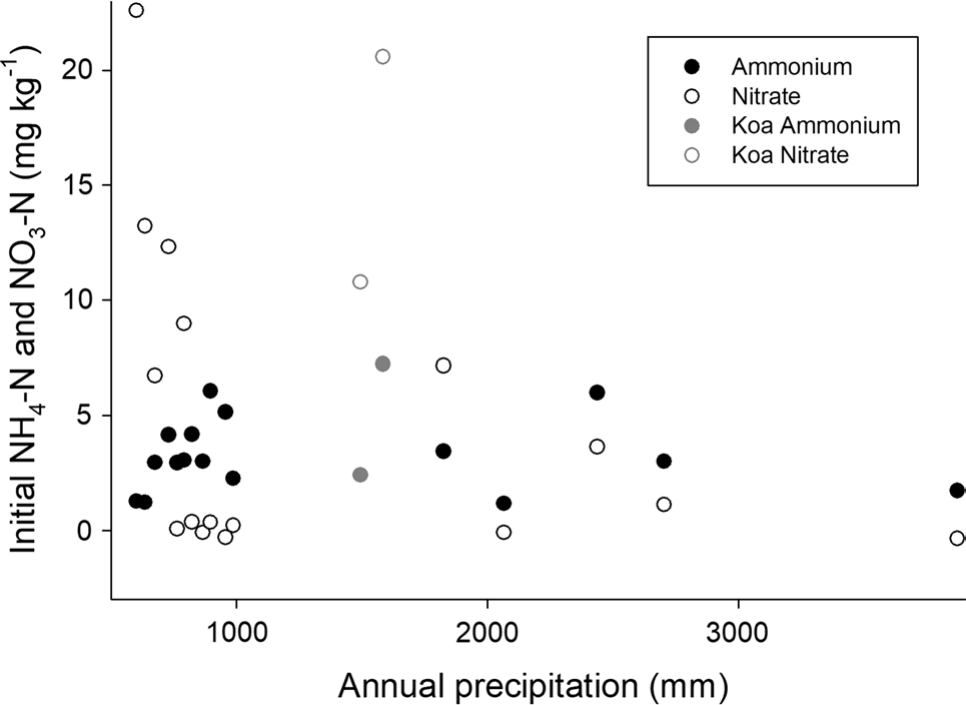
Initial ammonium-N and nitrate-N concentrations as a function of precipitation for sites across the Kaua’i climate gradient. Symbols as in Fig. 1

At the time samples were collected, pools of ammonium-N across the Kauai gradient did not vary substantially (Fig. 2), but pools of nitrate-N differed more than those of ammonium-N and in more interpretable ways. Pools of both ammonium and nitrate in soil are variable enough that one-time sampling does not generally characterize them well, but the nitrate concentrations in the driest sites here were sufficiently high to be interesting. The 4 lowest-rainfall sites (from 601 to 729 mm year^−1^ annual precipitation) averaged 13.7 mg NO_3_^−^N kg^−1^ soil (SE 3.3), while 5 sites that received from 822 to 987 mm year^−1^ annual precipitation averaged 0.10 mg NO_3_^−^N kg^−1^ soil (SE 0.15) (*P* < 0.01 by rank-sum test) (Fig. 2). Nitrate pools also were high in the two *A. koa*-dominated sites with high rates of mineralization and nitrification.

### Soil carbon/nitrogen ratio

**Fig. 3:**
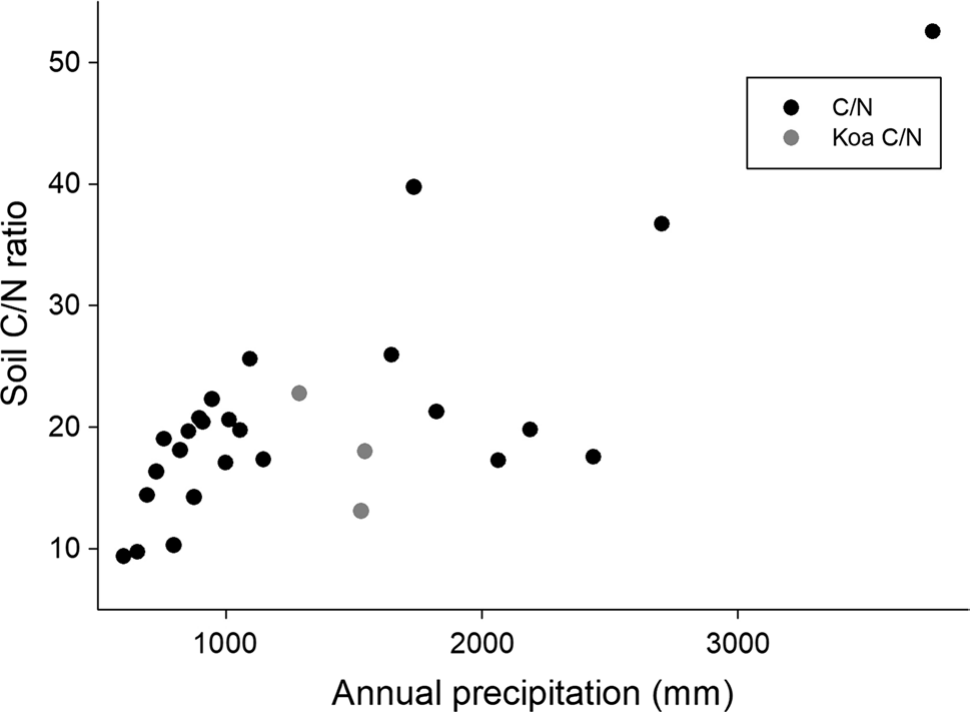
The ratio of total C to total N (on a mass basis) as a function of precipitation for sites across the Kaua’i climate gradient. Symbols as in Fig. 1

The C/N ratio in soils along the Kaua’i gradient increased from < 10 in the driest sites to > 50 in the wettest site (Fig. 3). The two *A. koa*-dominated sites with the highest rates of net N mineralization and nitrification had moderately low C/N ratios—higher than those in the driest sites but lower than slightly drier or wetter sites on the gradient.

### Soil δ^15^N natural abundance

**Fig. 4:**
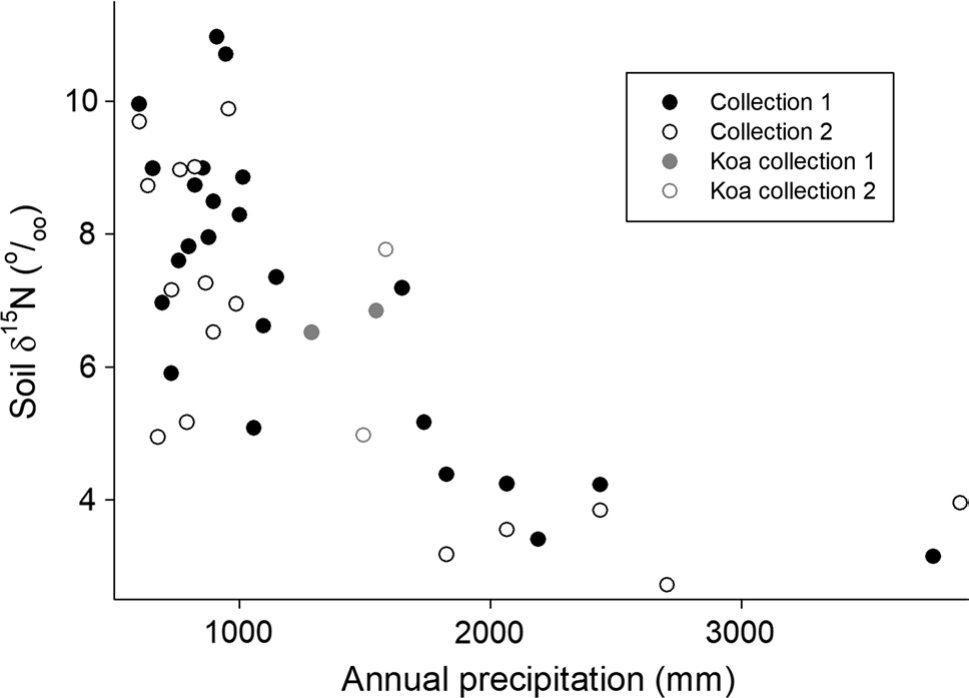
The δ^15^N (in ^ο^/_οο_) of total soil N as a function of precipitation, for two independent sets of soil samples collected across the Kaua’i climate gradient. Symbols as in Fig. 1

The relative abundance of the naturally occurring stable isotope ^15^N (δ^15^N) integrates multiple processes in the N cycle, often making it difficult to determine how particular processes affect variation in δ^15^N. On the Kaua’i climate gradient, this signal was relatively interpretable; the clearest pattern was an increasing relative abundance of ^15^N in drier sites on the gradient (Fig. 4). This increase was similar in the two independent collections, with a slope of − 0.0023 and a standard error of 0.0004 (*r*^2^ = 0.56, *P* < 0.001) for a regression of δ^15^N versus precipitation in mm for the first set collected in 2011 and a slope of − 0.0018 and standard error of 0.0005 (*r*^2^ = 0.48, *P* = 0.001) for the second set collected in 2017.

## Discussion

Our focus here was to evaluate whether pedogenic thresholds and soil process domains (Chadwick and Chorover 2001; Vitousek and Chadwick 2013) provide useful information for understanding N dynamics. Earlier, we had defined pedogenic thresholds on the Kaua’i gradient at ~ 900 and ~ 2500 mm year^−1^ annual precipitation, based entirely upon patterns in the pools and availability of rock-derived nutrients (Vitousek and Chadwick 2013) with no knowledge of N pools or processes. These thresholds bounded three domains: one below 900 mm year^−1^ precipitation, where weatherable minerals remained in the soil and rock-derived exchangeable cations were elevated; one between 900 and 2500 mm year^−1^ annual precipitation, where soils were acid and infertile; and one with > 2500 mm year^−1^ precipitation, where iron reduction and loss were substantial.

The mean values by domain for selected nitrogen pools, processes, and indicators are summarized in Table 1. In that table, we present results separately for sites dominated by the native N-fixing tree *Acacia koa*; in terms of soil process domains defined by rock-derived elements, these sites were within the middle (acid, infertile) soil process domain. Only two *koa*-dominated sites were sampled, along with only two sites in the wettest soil process domain, precluding statistical comparisons of those groups to others. Overall, only soil C/N ratios differed statistically (*P* < 0.01, *T* test with unequal variances) between the domain with ongoing weathering and the acid, infertile domain (domains 1 and 2). The domain dominated by iron reduction (domain 3) had very high C/N ratios, suggesting that the pattern of increasing C/N ratio with increasing precipitation continued into the wettest sites (as shown in Fig. 3). Soil C/N ratios in the *koa*-dominated stands were within the range of values observed in domain 2. Net N mineralization at both ambient and adjusted soil moisture did not differ statistically between domains 1 and 2; the limited number of sites sampled suggests that N mineralization was not different in domain 3 either, although N mineralization was strikingly elevated in the *Acacia koa*-dominated sites (Table 1; Fig. 1).

**Table 1:** Selected properties related to N pools and cycling, as a function of soil process domains on the Kaua’i climate gradient

	Ambient	Adjusted	Initial	δ^15^N	C/N
	Net N min	Net N min	Nitrate		
Domain 1 (< 900 mm year^−1^)	0.14 (0.06)	0.26 (0.12)	7.2 (2.8)	7.8 (0.3)	15.2 (1.4)
Domain 2 (900–2540 mm year^−1^)	0.32 (0.18)	0.33 (0.22)	2.1 (1.4)	6.3 (0.6)	21.9 (2)
Domain 3 (> 2540 mm year^−1^)	0.36	0.29	0.4	3.2	44.6
Dominated by *Acacia koa* (1250–1550 mm year^−1^)	1.4	1.4	15.7	6.5	18.0
Adjusted threshold					
Domain 1 (<800 mm year^−1^)	0.23 (0.06)	0.42 (0.11)	10.7 (3.1)	7.7 (0.5)	13.2 (1.6)
Domain 2 (800–2540 mm year^−1^)	0.09 (0.09)	0.05 (0.09)	1.1 (1.0)	7.2 (0.5)	19.6 (0.8)

Ambient and adjusted net N mineralization defined as described in the text. Mineralization in mg kg^−1^ d^−1^, initial nitrate (before incubation) in mg kg^−1^ of dry soil, δ^15^N in ^ο^/_οο_, and C/N ratio in terms of mass. Values are means, with standard errors in parentheses; there were only two sites in the wet domain and two *koa*-dominated sites, so only means are reported for those classes of sites. We also report means and standard errors for the first two domains with the boundary between them adjusted to 800 mm year^−1^ precipitation (instead of 900 mm year^−1^)

There is an element of arbitrariness in how we determined the location of the pedogenic thresholds that bound domains. Accordingly, we explored the consequences of shifting the boundary between domains 1 and 2 from 900 mm year^−1^ to 800 mm year^−1^ [within a reasonable range given the patterns in rock-derived elements (Vitousek and Chadwick 2013)]. With this adjustment (also summarized in Table 1), both net N mineralization at adjusted water content and initial nitrate pools differed significantly (*P* < 0.05, *T* test with unequal variances) between domains 1 and 2, and soil C/N ratios remained significantly different (*P* < 0.01).

**Fig. 5:**
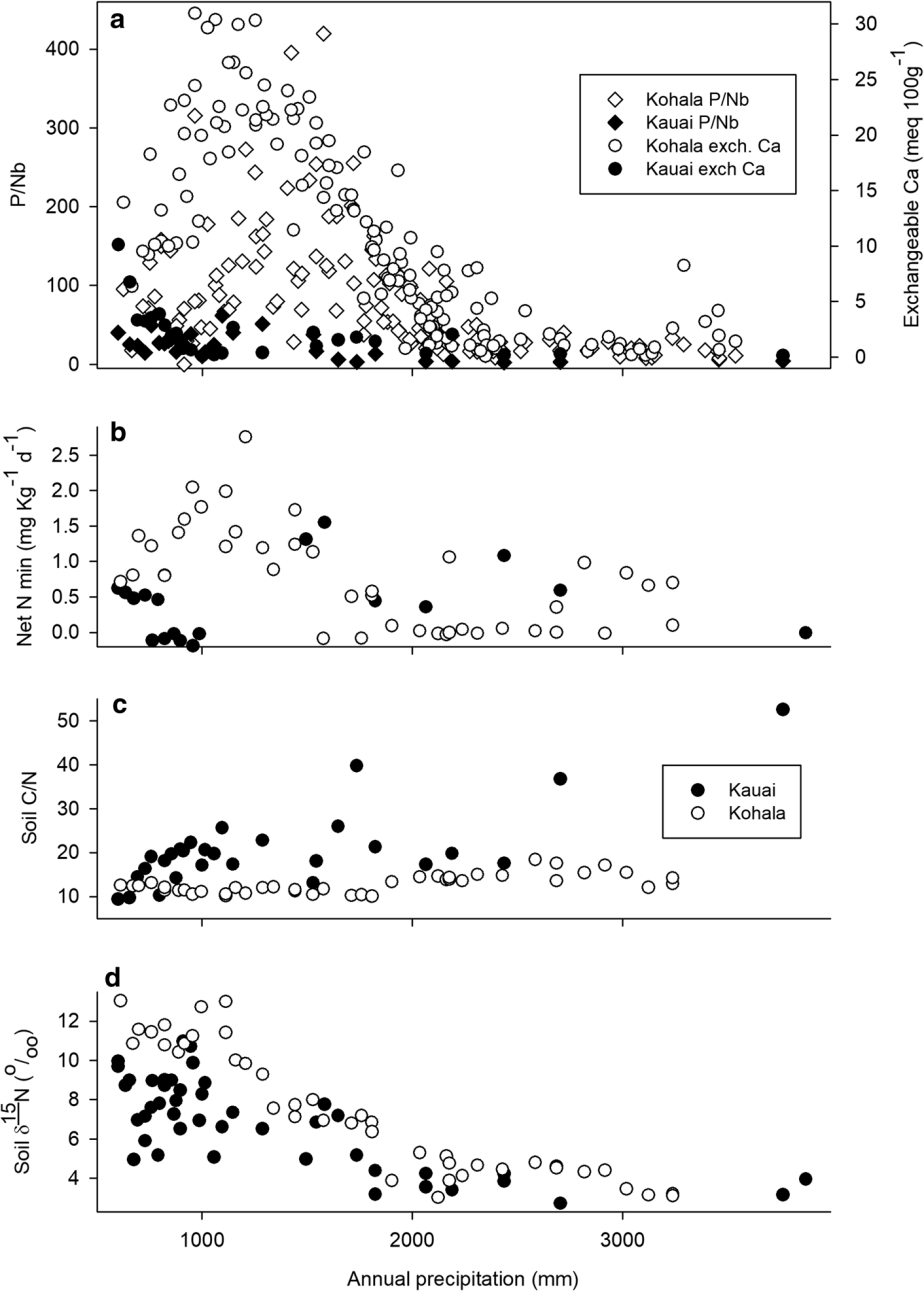
Comparison of soil properties across the Kaua’i and Kohala climate gradients, as a function of precipitation. All plots exclude sites on the Kohala gradient with less than 600 mm year^−1^ precipitation (the lowest value on the Kaua’i gradient). **a** Exchangeable Ca and total P/Nb ratios. P/Nb ratios in the nutrient-rich domain in Kohala are well in excess of those in parent material due to biological uplift of P and consequent enrichment of surface soils, as described in Vitousek and Chadwick (2013). **b** Net N mineralization in soils adjusted to a moisture content where soil water did not limit rates of N mineralization (45% on Kaua’i, 65% on Kohala); Kohala data from von Sperber et al. (2017). **c** Initial nitrate-N concentrations, Kohala data from von Sperber et al. (2017). **d** Soil δ^15^N, Kohala data from von Sperber et al. (2017)

In addition to evaluating pedogenic thresholds and soil process domains on the Kaua’i gradient, comparing Kaua’i to the Kohala gradient can yield insight into controls of N dynamics. We observed that the pools and availability of rock-derived nutrients were elevated in a soil process domain that occupied the middle portion of the Kohala gradient and that also occurred at the driest extreme of the Kaua’i gradient (Fig. 5a). Elevated pools of available forms of rock-derived nutrients were associated with a peak in N pools and transformations on the Kohala gradient (von Sperber et al. 2017). Moreover, pools of nitrate and potential rates of N transformations also were significantly elevated in that domain (compared to slightly wetter sites) on the Kaua’i gradient (Figs. 1, 2, 5b; Table 1), as would be expected if biological N fixers have an advantage where other resources are abundant (and where through their activity they increase the pools and transformation rates of N) (Vitousek and Field 1999; Menge et al. 2008). However, the highest rates of N mineralization on both gradients occurred at annual rainfall near 1500 mm (a water balance near 0), which is within the domain where rock-derived nutrients were abundant on Kohala but where on Kaua’i soils were highly infertile in terms of Ca and P pools and availability (Vitousek and Chadwick 2013). The native N-fixing tree *Acacia koa* dominated the sites with the highest rates of N mineralization on Kaua’i, in contrast to Kohala where *Acacia koa* was absent but where introduced plants with N-fixing symbioses were most abundant in the domain rich in rock-derived nutrients.

**Fig. 6:**
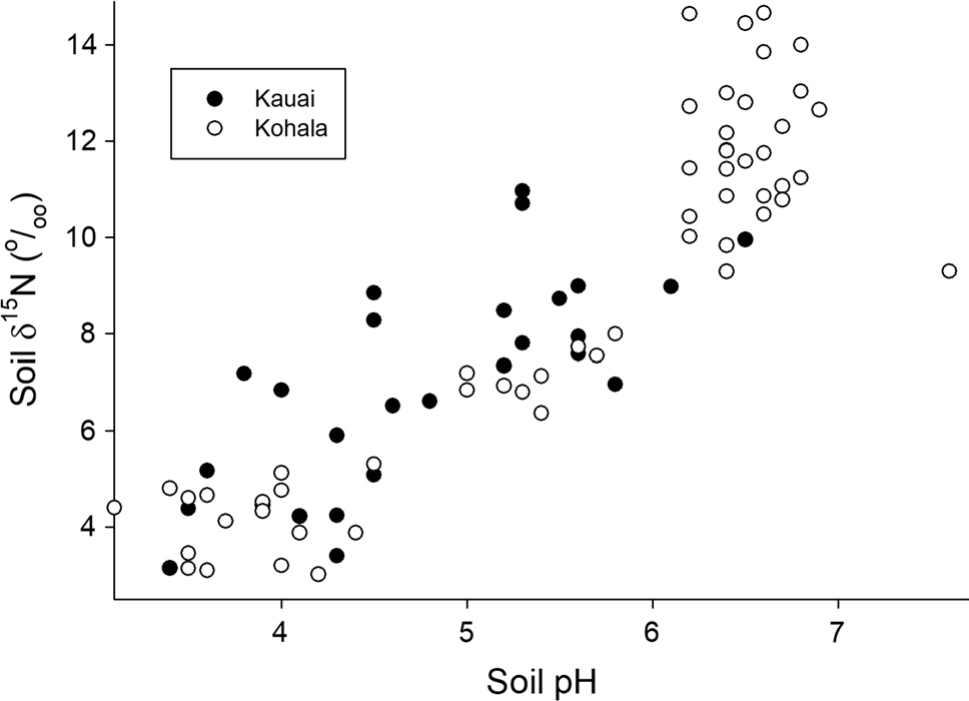
Relationship between soil pH and soil δ^15^N for both gradients. The Kohala gradient reaches to lower rainfall than the Kaua’i gradient, likely explaining the higher pH values in some sites there. Soil pH data from Vitousek and Chadwick (2013); Kohala δ^15^N from von Sperber et al. (2017)

The pattern of variation in soil δ^15^N was similar on both gradients. It increased (became more enriched in ^15^N) from wet to dry sites (Fig. 5d) and was strongly and significantly positively correlated with soil pH on both gradients (slope 1.9, standard error 0.35, *r*^2^ = 0.53, *P* < 0.001 for Kaua’i; slope 2.68, standard error 0.17, *r*^2^ = 0.83, *P* < 0.001 for Kohala) (Fig. 6). A progressive increase in δ^15^N in sites arrayed from wet to dry on climate gradients is widely observed (Austin and Sala 2002; Amundson et al. 2003; Wang et al. 2014; Craine et al. 2015; von Sperber et al. 2017); this pattern is believed to reflect a progressive increase in highly fractionating gaseous losses of N in progressively drier sites. The correlation with pH here suggests that ammonia volatilization (which strongly discriminates against the heavier ^15^N isotope, leaving residual soil N enriched in ^15^N) is a dominant process controlling the pattern. Denitrification also fractionates strongly against ^15^N—and it can be important in high-rainfall sites elsewhere in Hawai’i (Houlton et al. 2006)—but the positive correlation with pH here (and the similarity of the pattern on both gradients) suggests that ammonia volatilization controls the pattern.

In contrast, while soil C/N ratios (Fig. 5c) increased significantly (*P* < 0.001) from dry to wet on both gradients, the slope was more than 5× greater on Kaua’i (0.0017 for Kohala and 0.009 for Kaua’i. Soil C/N ratios in the high-rainfall soil process domain dominated by iron reduction and loss on Kaua’i (which did not occur on the younger gradient) were much higher than C/N ratios elsewhere.

We conclude that soil process domains are not always useful for evaluating N dynamics. On the Kaua’i gradient, the abundance of *A. koa* was more strongly controlled by climate than by soil process domains. While the highest rates of N mineralization and nitrification were associated with the abundance of plants with N-fixing symbioses on both gradients, their abundance was associated with a domain rich in rock-derived nutrients on the younger but not the older gradient. Biological invasion and/or land-use change could have eliminated other N fixers from drier sites where rock-derived nutrients are more available on the Kaua’i gradient, skewing our results. However, the extent to which the importance of N fixation is controlled directly by climate (peaking near a water balance of 0) rather than by soil process domains is worth exploring further.

## References

[CR1] Amundson R, Austin AT, Schuur EAG, Matzek V, Uebersax A, Brenner D, Baisden WT, Kendall C (2003). Global patterns of the isotopic composition of soil and plant nitrogen. Glob Biogeochem Cycles.

[CR2] Aplet GH, Hughes RF, Vitousek PM (1998). Ecosystem development on Hawaiian lava flows: biomass and species composition. J Veg Sci.

[CR3] Austin AT, Sala O (2002). Carbon and nitrogen dynamics across a natural precipitation gradient in Patagonia, Argentina. J Veg Sci.

[CR4] Chadwick OA, Chorover J (2001). The chemistry of pedogenic thresholds. Geoderma.

[CR5] Chadwick OA, Gavenda RT, Kelly EF, Ziegler K, Olson CG, Elliott WD, Hendricks DM (2003). The impact of climate on the biogeochemical functioning of volcanic soils. Chem Geol.

[CR6] Elser JJ, Bracken MES, Cleland EE, Gruner DS, Harpole WS, Hillebrand H, Ngai JT, Seabloom EW, Shurin JB, Smith JE (2007). Global analysis of nitrogen and phosphorus limitation of primary producers in freshwater, marine, and terrestrial ecoystems. Ecol Lett.

[CR7] Foote DE, Hil ELI, Nakamura S, Stephens F (1972). Soil survey of the Islands of Kauai, Oahu, Maui, Molokai, and Lanai, State of Hawaii.

[CR8] Garcia MO, Swinnard L, Weis D, Green AR, Tagami T, Sano H, Gandy CE (2010). Petrology, geochemistry, and geochronology of Kaua’i lavas over 4.5 Myr: implications for the origin of rejuvenated volcanism and the evolution of the Hawaiian plume. J. Petrology.

[CR9] Giambelluca TW, Chen Q, Frazier AG, Price JP, Chen Y-L, Chu P-S, Eischeid JK, Delparte DM (2013). Online rainfall atlas of Hawai’i. Bull Amer Meteor Soc.

[CR10] Giambelluca TW, Shuai X, Barnes ML, Alliss RJ, Longman RJ, Miura T, Chen Q, Frazier AG, Mudd RG, Cuo L, Businger AD (2014) Evapotranspiration of Hawai’i. Final report submitted to the US Army Corps of Engineers—Honolulu District, and the Commission on Water Resource Management, State of Hawai’i

[CR11] Harrington RA, Fownes JH, Vitousek PM (2001). Production and resource-use efficiencies in N- and P-limited tropical forest ecosystems. Ecosystems.

[CR12] Herbert DA, Fownes JH (1995). Phosphorus limitation of forest leaf area and net primary productivity on a weathered tropical soil. Biogeochemistry.

[CR13] Hogberg P, Nasholm T, Franklin O, Hogberg MN (2017). Tamm review: on the nature of nitrogen limitation to plant growth in Fennoscandian boreal forests. For Ecol Manag.

[CR14] Hotchkiss SC, Vitousek PM, Chadwick OA, Price JP (2000). Climate cycles, geomorphological change, and the interpretation of soil and ecosystem development. Ecosystems.

[CR15] Houlton BZ, Sigman D, Hedin LO (2006). Isotopic evidence for large gaseous nitrogen losses from tropical forests. PNAS.

[CR16] Kuo S, Sparks DL, Page AL, Helmke PA, Loeppert RH (1996). Phosphorus. SSSA book series, methods of soil analysis part 3—chemical methods 5.3.

[CR17] Lavkulich LM (1981). Methods manual: pedology laboratory.

[CR18] MacDonald GA, Abbott AT, Peterson FL (1983). Volcanoes in the sea: the geology of Hawaii (2nd Edition).

[CR19] Menge DNL, Levin SA, Hedin LO (2008). Evolutionary tradeoffs can select against nitrogen fixation and thereby maintain nitrogen limitation. Proc Natl Acad Sci USA.

[CR20] Muhs DR (1984). Intrinsic thresholds in soil systems. Phys Geogr.

[CR21] Porter SC (1979). Hawaiian glacial ages. Quat Res.

[CR22] Richter DD, Yaalon DH (2012). “The changing model of soil” revisited. Soil Sci Soc Am J.

[CR23] Torn MS, Trumbore SE, Chadwick OA, Vitousek PM, Hendricks DM (1997). Mineral control of soil carbon storage and turnover. Nature.

[CR24] Turner BL, Romero TE (2009). Short-term changes in extractable inorganic nutrients during storage of tropical rain forest soils. Soil Sci Soc Am J.

[CR25] Vitousek PM (2004). Nutrient cycling and limitation: Hawai’i as a model system.

[CR26] Vitousek PM, Chadwick OA (2013). Pedogenic thresholds and soil process domains in basalt-derived soils. Ecosystems.

[CR27] Vitousek PM, Field CB (1999). Ecosystem constraints to symbiotic nitrogen fixers: a simple model and its implications. Biogeochemistry.

[CR28] Vitousek PM, Aplet G, Turner DR, Lockwood JJ (1992). The Mauna Loa environmental matrix: foliar and soil nutrients. Oecologia.

[CR29] Vitousek PM, Ladefoged TL, Kirch PV, Hartshorn AS, Graves MW, Hotchkiss SC, Tuljapurkar S, Chadwick OA (2004). Agriculture, soils, and society in precontact Hawai’i. Science.

[CR30] Vitousek PM, Dixon JL, Chadwick OA (2016). Pedogenic thresholds on basalt and non-basalt soils: observations and a simple model. Biogeochemistry.

[CR31] von Sperber C, Chadwick OA, Casciotti KL, Peay KG, Francis CA, Kim AE, Vitousek PM (2017). Controls of nitrogen cycling evaluated along a well-characterized climate gradient. Ecology.

[CR32] Wang C, Wang X, Liu D, Wu H, Lu X, Fang Y, Cheng W, Luo W, Jiang P, Shi J, Yin H, Zhou J, Han X, Bai E (2014). Aridity threshold in controlling ecosystem nitrogen cycling in arid and semi-arid grasslands. Nature Comm.

